# Retraction Note to: Regulation of autophagy by the nuclear factor κB signaling pathway in the hippocampus of rats with sepsis

**DOI:** 10.1186/s12974-023-02726-9

**Published:** 2023-02-26

**Authors:** YunJie Su, Yi Qu, FengYan Zhao, HuaFeng Li, DeZhi Mu, XiHong Li

**Affiliations:** 1 Department of Pediatrics, West China Second University Hospital, Sichuan University, Chengdu, 610041 China; 2 Key Laboratory of Obstetric and Gynecologic and Pediatric Diseases and Birth Defects of Ministry of Education, Sichuan University, Chengdu, 610041 China; 3 Department of Pediatrics and Neurology, University of California, San Francisco, CA 94143 USA; 4 Department of Anesthesiology, West China Second University Hospital, Sichuan University, Chengdu, 610041 China

**Retraction Note to** **:** **Journal of Neuroinflammation (2015) 12:116** **https://doi.org/10.1186/s12974-015-0336-2**

The Editors-in-Chief have retracted this article. After publication, concerns were raised regarding Fig. 5:

• Figures 5A and 5B: the NF-kB panels appear to contain overlapping lanes.

• Figures 5A and 5B: the Actin panels appear to contain overlapping lanes.

The data reported in this article are therefore unreliable.

Corresponding author XiHong Li has stated that the authors agree with the retraction of the article but disagree with the wording of the retraction notice. Author Dezhi Mu has not explicitly stated whether they agree or disagree with the retraction. The other authors have not separately responded to correspondence regarding this retraction.

